# IGFBP2 Drives Regulatory T Cell Differentiation through STAT3/IDO Signaling Pathway in Pancreatic Cancer

**DOI:** 10.3390/jpm12122005

**Published:** 2022-12-03

**Authors:** Longhao Sun, Yang Zhang, Tiantian Yang, Junhang Chen, Xuebin Zhang, Xiaoyu Liang

**Affiliations:** 1 Department of General Surgery, Tianjin Medical University General Hospital, Tianjin 300052, China; 2 Department of Cancer Biology, Wake Forest School of Medicine, Winston Salem, NC 27157, USA; 3 Department of Pathology, The University of Texas MD Anderson Cancer Center, Houston, TX 77030, USA; 4 Department of Pathology, Tianjin Huanhu Hospital, Tianjin 300350, China

**Keywords:** insulin-like growth factor binding protein 2, regulatory T cells, pancreatic cancer, signal transducer and activator of transcription 3, indoleamine 2, 3-dioxygenase

## Abstract

Pancreatic ductal adenocarcinoma (PDAC) represents one of the deadliest malignancies. Elevated regulatory T cell (Treg) infiltration has a potent immunosuppressive function in tumor biology, which contributes to low survival in PDAC. Nonetheless, the crosstalk between malignant cells and tumor-infiltrating Tregs in PDAC is not well understood. Here, clinical data demonstrates that the insulin-like growth factor binding protein 2 (IGFBP2) is associated with Treg accumulation in the microenvironment of PDAC in humans. Additionally, IGFBP2 increases Treg infiltration in the tumor microenvironment and promotes disease progression in mouse PDAC. Bioinformatic analysis and mechanistic assessment reveals IGFBP2 upregulated indoleamine 2, 3-dioxygenase (IDO) by activating signal transducer and activator of transcription 3 (STAT3) signaling in PDAC cells, thus inducing Treg differentiation and an immunosuppressive tumor microenvironment. These findings provide mechanistic insights into an important molecular pathway that promotes an immunosuppressive microenvironment, which suggests the IGFBP2 axis as a potential target for improved immune response in PDAC.

## 1. Introduction

Pancreatic ductal adenocarcinoma (PDAC) is a devastating disease worldwide, whose global burden has significantly increased over the past 25 years [1,2]. PDAC still has the worst prognosis among malignancies, with a 5-year survival below 8% [3,4]. Despite important efforts in developing new therapies, no substantial improvement has been achieved in patient survival in the last decades [5,6]. It is critical to understand the unique underlying biological mechanism of PDAC and to identify novel treatment modalities [7].

One of the hallmarks of PDAC is that it is full of stroma in its tumor microenvironment (TME) [8,9]. The cellular stroma constituents, especially infiltrating immune cells, contribute to virtually all biological events in PDAC and participate in hypoxia and immunosuppressive TME [10,11,12]. Regulatory T cells (Tregs) are a classic immunosuppressive T cell subset, which accumulate at the tumor site, secrete various cytokines to inhibit T cell cytotoxicity, and mediate tumor cell evasion of immune surveillance. As key tumor-infiltrating immune cells in PDAC, Tregs induce tumorigenic, angiogenic, and metastatic mechanisms, as well as resistance to chemotherapy. Increased Treg infiltration constitutes a key inducer of immune tolerance, which reduces patient survival in PDAC [13,14].

Insulin-like growth factor-binding protein 2 (IGFBP2) is considered a potent oncogene in many solid tumors [15]. We recently demonstrated that IGFBP2 regulates epithelial-to-mesenchymal transition (EMT) and metastasis via NF-κB signaling. In addition, IGFBP2 increased the nuclear amounts of the epidermal growth factor receptor (EGFR) to induce signal transducer and activator of transcription 3 (STAT3) that upregulates cyclooxygenase-2 (COX-2), which contributed to the generation of an immunosuppressive TME [16]. It was suggested that IGFBP2 contributes to immune events in PDAC because it induces tumor progression via the enhancement of alternative macrophage polarization by regulating STAT3 signaling [15].

Continuous STAT3 induction is considered an important regulator of PDAC tumorigenesis, acinar-ductal-metaplasia (ADM) generation, disease progression, apoptosis, and inflammation suppression. STAT3 is also involved in the crosstalk between the TME and the immune system, promoting immune evasion of cancer cells [17,18]. Induced STAT3 in cancer results in the upregulation of specific cytokines that in turn activate STAT3 in multiple immune cells such as Tregs. Enhanced STAT3 activation in immune cells induces diverse immunosuppressive events and impairs antitumor immunity [19]. Specifically, STAT3 controls the expression of indoleamine 2, 3-dioxygenase (IDO), which inhibits T cell proliferation and induces Treg differentiation by metabolizing tryptophan to produce kynurenine [20].

In this study, we provide evidence that IGFBP2 increases Treg recruitment and tumor progression in PDAC patients. We further show that IGFBP2 augments IDO expression through the STAT3 signaling pathway in PDAC, thereby inducing Treg differentiation and tumor growth. 

## 2. Materials and Methods

### 2.1. Patients and Tissue Samples

The utilization of clinical samples had approval from the institutional research ethics committee of Tianjin Medical University General Hospital (TMUGH). Patient tissue samples were collected from 50 patients surgically treated in the aforementioned hospital from 2018 to 2021, who were histologically diagnosed with PDAC. After receiving informed consent, clinicodemographic features were examined. 

### 2.2. Immunohistochemical Analysis, Flow Cytometry and Enzyme-Linked Immunosorbent Assay

Consecutive formalin-fixed, paraffin-embedded tumor sections underwent immunohistochemical analysis (IHC) for IGFBP2, FOXP3, and IDO (no. sc-130070, sc-53876, and sc-53978; Santa Cruz Biotechnology, Dallas, TX, USA). The results were scored as described previously [21] by two pathologists blinded to clinical and pathological findings. Meanwhile, freshly collected PDAC surgical specimens were treated with collagenase (1 mg/mL), hyaluronidase (2.5 U/mL), and DNase (0.1 mg/mL) to generate single-cell suspensions that were analyzed flow-cytometrically for IDO, CD4, CD25, FOXP3, CD8, and CD45 expression on tumor cells (no. 567867, 641398, 567488, 561493, 347313, and 348795; BD Biosciences, San Jose, CA, USA). Paired adjacent non-cancerous specimens were also obtained for IGFBP2 detection by IHC. The remaining tissues from the freshly collected patient samples were weighed and homogenized for 5 min with a bead homogenizer in PBS (1 mass: 3 vol), followed by a 20 min centrifugation at 10,000× *g* and 4 °C. Then, enzyme-linked immunosorbent assay (ELISA) was performed to measure the concentrations of tryptophan (no. KA1916; Novus Biologicals, Littleton, CO, USA) and kynurenine (no. MBS495082; MyBioSource, San Diego, CA, USA) in supernatants. Results were compared between high and low IGFBP2 expression groups, which were determined by IHC.

### 2.3. Patient-Derived Xenograft Cells and Cell Culture

Human PDAC PDX MDA-PATC53 and MDA-PATC148 (all KRas G12D) cell lines with elevated and low endogenous IGFBP2 amounts, respectively, established as described in a previous report, underwent culture in Dulbecco’s Modified Eagle Medium/F-12 medium containing 10% fetal bovine serum (FBS) [15,16,22]. Authenticated mouse PDAC Panc02 cells were provided by the ATCC (Rockville).

### 2.4. Orthotopic PDAC Mouse Model

Female specific pathogen-free (SPF) C57BL/6 mice (age, 4 weeks; weight, 20–22 g) were used in these experiments after approval from the Ethics Committee of TMUGH, following the NIH Guide for the Care and Use of Laboratory Animals. Mice were housed with free access to food and water under the standard 12 h light/dark cycles at room temperature (22–24 °C) and 40–60% humidity. Panc02 cells stably overexpressing IGFBP2 were generated by infection with mouse IGFBP2 lentiviruses (no. NM_001313992.1; GeneCopoeia, Portland, OR, USA) with polybrene and subsequent culture under puromycin pressure for 3 weeks. Forty-six mice were randomly assigned to the Panc02-EV and Panc02-IGFBP2 groups. Under aseptic conditions, mice were anesthetized, and a left paramedian abdominal incision was made [23]. Then, 2 × 10^6^ Panc02-EV or Panc02-IGFBP2 cells resuspended in 20 µL PBS (1 × 10^7^ cells/mL) were inoculated directly into the pancreatic parenchyma for establishing the orthotopic murine model of PDAC. Peritoneum and skin were sutured using an absorbable suture. Wound healing, body weight, behavior, and physical condition of the mice were monitored at least twice a week over the total experimental time. The maximum diameter of the tumors allowed growing in the mice before euthanasia was 1.2 cm [24]. After 4 weeks, 8 mice from each group were randomly selected, and euthanasia was performed via cervical dislocation. Death was confirmed by cardiac and respiratory arrest. Tumor area was commonly separated from the adjacent non-cancerous area by the morphological character and hardness. Then xenograft tumors were extracted, weighed, and homogenized to prepare single-cell suspensions as described above for human samples for flow-cytometry detection of mouse Tregs (CD4^+^CD25^+^FOXP3^+^) and cytotoxic T cells (CD8^+^CD45^+^) (BD Biosciences). For the determination of the liver metastatic foci number, the whole livers were harvested and immediately fixed in neutral formalin 10%. After tissues were paraffinized, serial sections were prepared. Metastatic foci, which are macroscopically visible to the naked eye as white tumors, were counted. Overall survival of other mice was presented by Kaplan–Meier curves.

### 2.5. Lentivirus, Plasmids, and siRNAs 

Stable IGFBP2 and IDO overexpression cell lines (MDA-PATC148BP2 and MDA-PATC148IDO) were generated by infecting MDA-PATC148 cells with human IGFBP2 and IDO lentiviral particles (GeneCopoeia), respectively, utilizing polybrene, with subsequent incubation under puromycin pressure for 3 weeks. 

The Y705-mutated STAT3 sequence was obtained by mutating the nucleotide sequence encoding the Y705 of STAT3 (NM_139276.2) into F705 with the QuikChange Lightning site-directed mutagenesis kit (Agilent Technologies, Clara, CA, USA). Human wildtype and mutant *STAT3* sequences underwent cloning into pcDNA3.1 plasmid (Invitrogen, Waltham, MA, USA), with verification by DNA sequencing. MDA-PATC148 cells were plated in 6-well plates at a density of 5 × 10^5^ cells/well in medium containing 10% FBS and cultured until 80% confluent. Cells were transfected with 4 µg plasmids with FuGENE HD (Promega) for 48 h. Stable cell lines were selected under G418 pressure for 3 weeks.

MDA-PATC53 cells (high endogenous IGFBP2) were transfected with 50 nM IDO1 (no. NM_002164; SASI_Hs01_00237226 or SASI_Hs01_00237227), STAT3 (no. NM_139276; SASI_Hs01_00061860 or SASI_Hs01_00061861), IGFBP2 (no. NM_000597; SASI_Hs02_00302878 or SASI_Hs01_00039595), (Supplemental Table S1), and scramble control siRNAs (no. SIC002; Sigma, St. Louis, MI, USA), respectively, with Lipofectamine RNAiMAX (no. 13778075; Invitrogen) as described above. MDA-PATC148 BP2 cells (elevated endogenous IGFBP2) also underwent transfection with STAT3 and scramble control siRNAs, respectively. After 48 h, total RNA and total protein were obtained for qRT-PCR and immunoblot, respectively.

### 2.6. T Cell Studies

Human CD8^+^ and CD4^+^ T cells, respectively obtained from PBMCs isolated from healthy blood donors utilizing CD8 and CD4 microbeads (no. 130-045-201 and 130-045-101; Miltenyi Biotech, Cologne, Germany), underwent culture with Dynabeads^®^ Human T-Activator CD3/CD28 (no. 11131D; Thermo-Fisher, Waltham, MA, USA) as directed by the manufacturer. Activated T cells were used in the following studies.

#### 2.6.1. Treg Induction and T Cell Suppression 

Activated CD4^+^ T cells underwent culture in a medium containing 100 pg/mL IL-2 (no. AF-200-02-10; Peprotech, London, UK) without (negative control) or with 10 ng/mL TGF-β (no. AF-100-21C-2; Peprotech; positive control), or they were co-cultured with PDAC cell lines differentially expressing IGFBP2 or IDO for 5 days. Suspension CD4^+^ cells were then assessed flow-cytometrically for FOXP3 expression as a measure of Treg induction. Tregs inducted by TGF-β or PDAC cell lines mentioned above were isolated by fluorescence-activated cell sorting and co-cultured with activated CD8^+^ T cells at the ratio of 1:2 in media containing L-2 for 2 days. Then CD8^+^ T cells were sorted by CD8 microbeads (Miltenyi Biotech) and stained with Ki-67 antibody. The mean inhibition ratio of Tregs to the proliferation of CD8^+^ T cells was calculated after the flow cytometry of the Ki-67 expression.

#### 2.6.2. T Cell Apoptosis Assay 

Activated CD8^+^ T cells underwent co-culture (1:1) with PDAC cell lines differentially expressing IGFBP2 or IDO for 8 h, after which the suspension T cells underwent purification using CD8 microbeads and were flow-cytometrically assessed with the Apoptosis Detection Kit (no. 556570; BD Pharmingen, San Diego, CA, USA), as directed by the manufacturer.

#### 2.6.3. Cytotoxicity Assay 

Activated CD8^+^ T cells underwent co-culture (1:1) for 8 h with PDAC cell lines differentially expressing IGFBP2 or IDO in media containing 100 pg/mL IL-2 (Peprotech) and were purified using CD8 microbeads (Miltenyi Biotech). MDA-PATC124 cells were co-cultured with experimentally treated CD8^+^ T cells, with an effector (T cells):target (MDA-PATC124 cells) ratio of 10:1 for 24 h. The percentage of 7-AAD^+^ PDAC cells was assessed flow-cytometrically as cell death rate.

#### 2.6.4. T Cell Proliferation and Cell Cycle Analysis 

Activated CD8^+^ T cells underwent co-culture with MDA-PATC53 or MDA-PATC148 cell lines differentially expressing IGFBP2 or IDO in media containing 100 pg/mL IL-2 (Peprotech) for 2 days. T cell purification utilized CD8 microbeads (Miltenyi Biotech), and T cell proliferation was examined by cell counting. Next, T cells were stained with Ki-67 antibody and PI for detecting Ki-67 positivity rate and cell cycle distribution by flow cytometry, respectively.

### 2.7. qRT-PCR

Total RNA extraction utilized the mirVana miRNA Isolation Kit (no. AM1561; Ambion). Reverse transcription utilized SuperScript II Reverse Transcriptase (no. 18064014; Invitrogen). TaqMan Gene Expression Assays (no. 4448490; Applied Biosciences, Beverly Hills, CA, USA) were utilized for quantitating human IGFBP2, IDO1, and STAT3 mRNA amounts by the 2^−ΔΔCt^ method, with GAPDH as a reference gene. Triplicate assays were carried out.

### 2.8. Immunoblot

Cell lysis was performed with the RIPA Buffer containing the Halt Phosphatase Inhibitor Cocktail (no. 78440; Thermo Fisher Scientific (Waltham, MA, USA)). Total protein underwent separation by 10% SDS-PAGE, followed by transfer onto nitrocellulose membranes. The membranes were successively incubated with anti-IGFBP2, anti-IDO, anti-STAT3, and anti-pSTAT3 (no. sc-25285, sc-53978, sc-8019, and sc-8059; Santa Cruz Biotechnology) primary antibodies, respectively, and HRP-linked secondary antibodies. SuperSignal West Pico chemiluminescent substrate (no. 24577; Thermo Fisher Scientific) was utilized for visualization.

### 2.9. Bioinformatic Analysis

The PDAC Australia (PDAC-AU) cohort of the International Cancer Genome Consortium (ICGC, https://dcc.icgc.org, accessed on 21 November 2021) comprises 461 specimens from 391 patient donors, including 91 specimens with reported RNA-seq and survival data (Supplemental Table S2). The patients were 47 men, 43 women, and 1 person with no disclosed gender information, and all were 36–86 years old. They were assigned to 3 groups according to STAT3 amounts, including the high- (top 20%), low- (bottom 20%), and intermediate-STAT3 (the remaining 60%) groups. Genes with differential expression between the high- and low-STAT3 groups were detected. The associations of STAT3 with IDO levels were assessed.

### 2.10. Statistical Analysis 

Data are mean ± SD and were compared by the Student’s *t*-test and analysis of variance (ANOVA) for group pairs and multiple groups, respectively. *p* < 0.05 indicated statistical significance. SPSS 17.0 (SPSS, USA) and R were utilized to analyze all data.

## 3. Results

### 3.1. IGFBP2 Overexpression Correlates with the Infiltration of Tregs in the Tumor Microenvironment and Tumor Progression in Clinical PDAC

**Figure 1:**
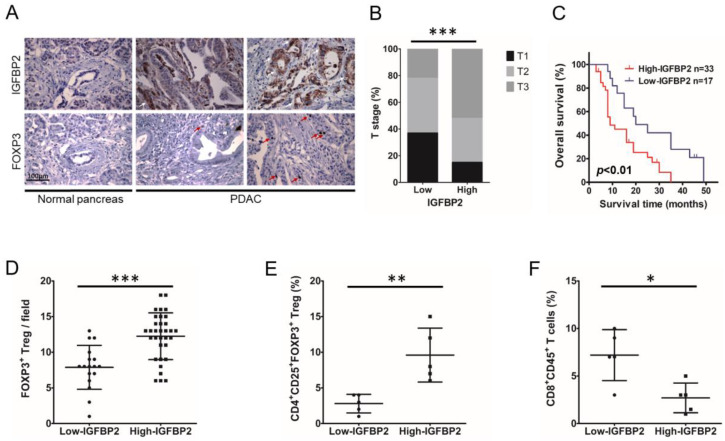
IGFBP2 overexpression is associated with Treg infiltration in the tumor microenvironment and disease progression in human PDAC. (**A**) IGFBP2 and FOXP3 amounts in PDAC and noncancerous pancreatic tissue specimens. Magnification: 400×. White arrows point to IGFBP2^+^ PDAC cells; red arrows point to FOXP3^+^ cells as Tregs. (**B**) Distributions of T stage in the high- and low-IGFBP2 expression groups (*n* = 50; *** *p* < 0.001 by χ^2^ test). (**C**) Kaplan–Meier curves comparing overall survival rates in PDAC cases with high and low IGFBP2 levels (*n* = 50; *p* < 0.01 by log-rank test). (**D**) Counts of FOXP3^+^ cells as Tregs in the PDAC microenvironment with reduced and elevated IGFBP2 amounts (*n* = 50; *** *p* < 0.001 by the Student’s *t*-test). (**E**) CD4^+^CD25^+^FOXP3^+^ cells (Tregs) assessed flow-cytometrically in PDAC tissue samples from freshly collected surgical specimens with reduced and elevated IGFBP2 amounts (*n* = 10; ** *p* < 0.01 by the Student’s *t*-test). (**F**) CD8^+^CD45^+^ T cells assessed flow-cytometrically in the latter PDAC tissue specimens (*n* = 10; * *p* < 0.05 by the Student’s *t*-test). Data are mean ± SD from 3 or more assays performed independently.

To examine IGFBP2′s role in PDAC progression, the correlation between IGFBP2 expression and PDAC progression was first evaluated in 50 patients treated in TMUGH. IHC demonstrated IGFBP2 (white arrows point to IGFBP2^+^ PDAC cells) was highly expressed in human PDAC but barely detected in adjacent noncancerous pancreatic cells (Figure 1A). Clinicopathological assessment demonstrated tumor IGFBP2 expression had a significant correlation with T stage. Individuals highly expressing IGFBP2 (++/+++) had increased tumor volumes and more advanced T stage compared with the low IGFBP2 group (−/+) (Figure 1B). Kaplan–Meier analysis suggested cases with tumors showing elevated IGFBP2 protein amounts had markedly reduced overall survival (OS) (*p* < 0.01; Figure 1C), indicating IGFBP2′s association with poor survival in PDAC cases.

To explore the association of IGFBP2 expression with Treg infiltration in the human PDAC microenvironment, IHC was first performed to detect FOXP3^+^ T cells (red arrows point to FOXP3+ cells as Tregs) in the above 50 PDAC specimens. The results revealed PDAC highly expressing IGFBP2 had markedly larger amounts of Tregs compared with the low IGFBP2 group (*p* < 0.001; Figure 1D). To validate the association of IGFBP2 with immune cell infiltration in PDAC, 10 freshly collected human PDAC specimens (5 low IGFBP2 and 5 high IGFBP2 cases determined by IHC based on non-cancerous specimens) were assessed flow-cytometrically for CD4^+^CD25^+^FOXP3^+^ (Tregs) and CD8^+^CD45^+^ (cytotoxic T cells). The high IGFBP2 group had markedly elevated percentages of Tregs (*p* < 0.01; Figure 1E), corroborating the above IHC data in a larger population (Figure 1A). Additionally, cytotoxic T cell rates were starkly decreased in the high IGFBP2 group (*p* < 0.05; Figure 1F). The above findings suggested elevated IGFBP2 amounts in PDAC were associated with increased infiltration of Tregs, suggesting an immunosuppressive TME and disease progression.

### 3.2. IGFBP2 Increases the Infiltration of Tregs in the TME and Promotes Disease Progression in Mouse PDAC

**Figure 2:**
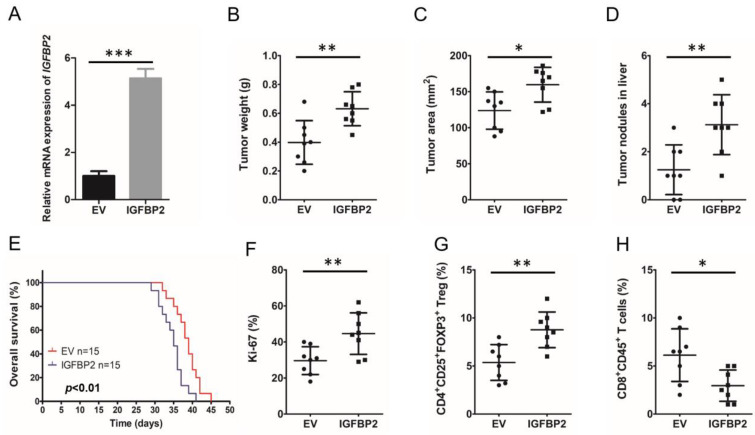
IGFBP2 increases Treg infiltration in the tumor microenvironment and promotes disease progression in mouse PDAC. (**A**) Panc02 cells stably overexpressing IGFBP2 were generated. *IGFBP2* mRNA amounts were assessed by qRT-PCR, normalized to GAPDH (*** *p* < 0.001 by Student’s *t*-test). C57BL/6 mice were used for establishing an orthotropic PDAC model. The animals were divided into two groups and injected with IGFBP2-overexpressing (IGFBP2) or empty vector-transfected control (EV) Panc02 cells. Following 4 weeks, euthanasia was performed, and tumor extraction was carried out. Tumor weights (**B**), tumor areas (**C**), and tumor nodules in the liver (**D**) for both mouse groups are shown (*n* = 8/group; * *p* < 0.05 ** *p* < 0.01 by the Student’s *t*-test). (**E**) The same orthotropic pancreatic carcinoma model was repeated, and the overall survival rates were analyzed (*n* = 15/group; *p* < 0.01 by log-rank test). (**F**) Flow cytometry analysis of Ki-67 in tumor cells from both groups of C57BL/6 mice (*n* = 8 per group; ** *p* < 0.01 by the Student’s *t*-test). CD4^+^CD25^+^FOXP3^+^ Tregs (**G**) and CD8^+^CD45^+^ T cells (**H**) in the PDAC microenvironment in both mouse groups, assessed flow-cytometrically, are shown (*n* = 8/group; * *p* < 0.05 ** *p* < 0.01 by the Student’s *t*-test). Data are mean ± SD from 3 or more assays performed independently.

To confirm IGFBP2 induces immunosuppressive mechanisms and tumor progression in vivo, an orthotopic PDAC mouse model was generated by injecting Panc02 cells harboring plasmids overexpressing mouse IGFBP2 (Panc02-IGFBP2) and the empty vector (Panc02-EV) into C57BL/6 mice, respectively (*n* = 8 per group). Before the mouse model was established, *IGFBP2* mRNA expression levels of Panc02-IGFBP2 and Panc02-EV were assessed. QRT-PCR revealed significantly upregulated IGFBP2 expression in Panc02-IGFBP2 (Figure 2A).

Four weekslater, euthanasia was carried out, followed by tumor extraction. The results revealed Panc02-IGFBP2 tumors were markedly heavier (*p* < 0.01) and larger (*p* < 0.05) than those of the Panc02-EV group (Figure 2B,C). The Panc02-IGFBP2 group also showed a higher number of hepatic metastatic foci compared with the Panc02-EV group (*p* < 0.01; Figure 2D). High IGFBP2 expression was also associated with markedly decreased OS in an independent mouse population (*n* = 15/group) (*p* < 0.01; Figure 2E). We next performed flow cytometry for detecting Ki-67 positive cells in fresh tissue, and Panc02-IGFBP2 tumors had a higher proliferation rate than Panic02-EV tumors (*p* < 0.01; Figure 2F). 

To examine the association of IGFBP2 expression with T cell infiltration in the TME in PDAC, cells from freshly collected mouse tumors were evaluated flow-cytometrically. Panc02-IGFBP2 tumors showed higher Treg (*p* < 0.01) and lower cytotoxic T cell (*p* < 0.05) rates compared with the Panc02-EV group; Figure 2G,H). The above data demonstrated roles for IGFBP2 in Treg accumulation and PDAC progression. We hypothesized that IGFBP2 promotes PDAC progression by altering T cell differentiation.

### 3.3. IGFBP2 Modifies T Cell Differentiation to an Immunosuppressive Phenotype

**Figure 3:**
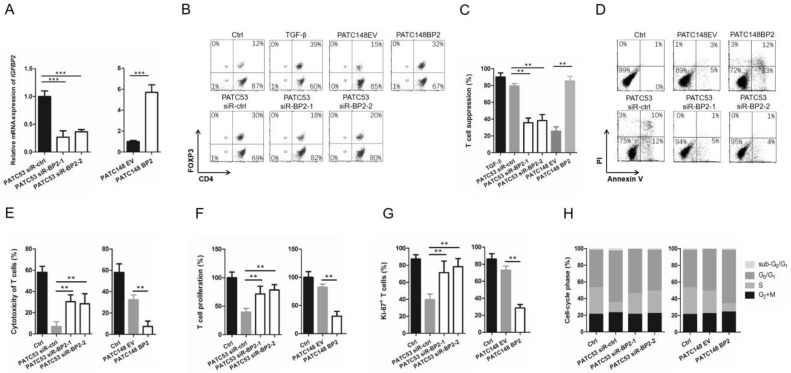
IGFBP2 alters T cell differentiation to promote an immunosuppressive phenotype. (**A**) Relative *IGFBP2* mRNA expression levels of different PDAC cell lines were assessed by qRT-PCR, normalized to *GAPDH* (*** *p* < 0.001 by ANOVA for PATC53 or the Student’s *t*-test for PTAC148). (**B**) Flow cytometry analysis of human CD4^+^ T cells cultured without or with TGF-β, or co-cultured with PDAC cells with differential expression of IGFBP2 for CD4^+^FOXP3^+^ Tregs. (**C**) Activated CD8^+^ T cells were co-cultured with the Tregs inducted by TGF-β or PDAC cell lines to verify their T cell suppressive function (** *p* < 0.01 by ANOVA for PATC53 or the Student’s *t*-test for PTAC148). (**D**) Human CD8^+^ T cells co-cultured with PDAC cells with differential expression of IGFBP2 assessed flow-cytometrically for apoptotic CD8^+^ T cells. (**E**–**H**) Cytotoxicity, proliferation, Ki-67, and cell cycle analyses were performed on the same CD8^+^ T cells described in (**D**) (** *p* < 0.01 by ANOVA for PATC53 or the Student’s *t*-test for PTAC148). Data are mean ± SD from 3 or more assays performed independently.

To assess IGFBP2′s effect on T cell differentiation, whether IGFBP2 induces Tregs in PDAC was first examined. Here, we established 2 pairs of patient-derived PDAC cell lines with a differential expression of IGFBP2. IGFBP2 was overexpressed in MDA-PATC148 cells (low endogenous IGFBP2) and knocked down in MDA-PATC53 cells (high endogenous IGFBP2) with siR-BP2-1 and -2 (SASI_Hs02_00302878 and SASI_Hs01_00039595). QRT-PCR revealed significantly decreased IGFBP2 expression in MDA-PATC53 siR-BP2-1 and -2 after siRNA transfection. Furthermore, upregulated IGFBP2 expression was detected in PATC148 BP2 than PATC148 EV (Figure 3A).

CD4^+^ T cells obtained from human PBMCs were activated and co-cultured with patient-derived PDAC cell lines with modulated IGFBP2 expression. Stimulation with TGF-β was performed in the positive control group. Following co-culture treatment, suspension T cells were flow-cytometrically evaluated for the CD4^+^FOXP3^+^ Treg population. Our data revealed that MDA-PATC148 BP2 cells (IGFBP2 overexpression) had a higher percentage of differentiated Tregs compared with MDA-PATC148 EV cells (endogenously low IGFBP2). In contrast, both MDA-PATC53 siRNA IGFBP2 knockdown cells (MDA-PATC53 siR-BP2-1 and -2) had lower percentages of Tregs than MDA-PATC53 siR-ctrl cells (endogenously high IGFBP2) (Figure 3B). 

Then, we verified the T cell suppressive function of Tregs inducted by TGF-β or PDAC cell lines mentioned above. The results revealed that TGF-β inducted Tregs showed a prominent T cell suppressive function as a positive control. Tregs induced by IGFBP2 silenced (MDA-PATC53 siR-1 and -2) PDAC cell significantly impaired the T cell suppressive function in comparison with Tregs induced MDA-PATC53 siR-ctrl cells with high endogenous IGFBP2. Conversely, MDA-PATC148 BP2 cell-induced Tregs showed stronger T cell suppressive function, compared with Tregs induced by MDA-PATC148 EV cells endogenously expressing low IGFBP2 (Figure 3C).

We further investigated whether differential expression of IGFBP2 in PDAC cells affects CD8^+^ T cell apoptosis, cytotoxicity, or proliferation. CD8^+^ T cells obtained from PMBCs were activated and then co-cultured with the PDAC cell lines described above at a ratio of 1:1. The results revealed that MDA-PATC148 BP2 cells impaired the cytotoxicity of T cells by promoting T cell apoptosis and reducing T cell proliferation via G1 arrest, compared with MDA-PATC148 EV cells endogenously expressing low IGFBP2 amounts. Conversely, IGFBP2 silencing (MDA-PATC53 siR-BP2-1 and -2) in PDAC cells significantly increased the amounts of cytotoxic T cells and suppressed apoptosis, in comparison with MDA-PATC53 siR-ctrl cells with high endogenous IGFBP2 (*p* < 0.01; Figure 3D–H). Jointly, these observations indicated that high IGFBP2 expression by PDAC tumor cells induced T cell differentiation to promote an immunosuppressive TME, leading to higher amounts of Tregs and impaired cytotoxicity and proliferation of cytotoxic T cells by inducing apoptosis.

### 3.4. IGFBP2 Activates STAT3 and Induces IDO Expression in Human PDAC

**Figure 4:**
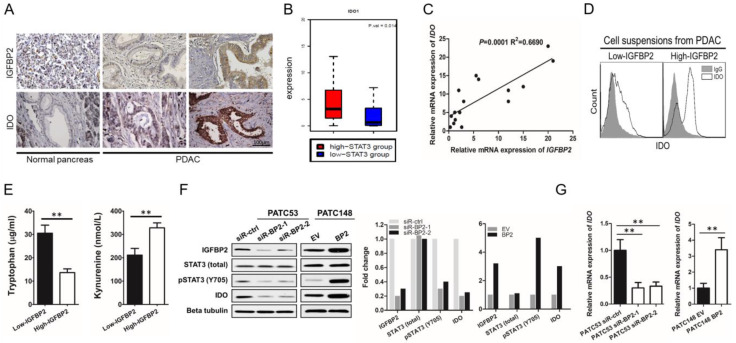
IGFBP2 activates STAT3 and increases IDO expression in human PDAC cells. (**A**) IGFBP2 and IDO amounts in PDAC and noncancerous pancreatic tissue specimens. Magnification: 400×. (**B**) IDO amounts in the high- and low-STAT3 groups of specimens in the ICGC PDAC-AU database (*n* = 91; *p* = 0.014 by the Wilcoxon rank sum test). (**C**) Correlation between IDO and IGFBP2 expression for each PDAC tissue from freshly collected surgical specimens, assessed by linear regression. (**D**) IDO^+^ cells in PDAC tissue specimens with low- and high-IGFBP2 expression, assessed flow-cytometrically. (**E**) Levels of tryptophan and L-kynurenine in PDAC tissue samples evaluated by ELISA (*n* = 16; ** *p* < 0.01 by the Student’s t-test). (**F**) Immunoblot of MDA-PATC53 cells after transfection with negative control (siR-ctrl) and IGFBP2 (GFBP2 and -2) siRNAs IGFBP2, respectively (left panel). Immunoblot of MDA-PATC148 cells upon transfection with negative control (EV) and human IGFBP2 (BP2) lentiviruses for IGFBP2 overexpression (right panel). (**G**) IDO mRNA amounts were assessed by qRT-PCR, normalized to GAPDH (** *p* < 0.01 by ANOVA for PATC53 or the Student’s t-test for PTAC148). Data are mean ± SD from 3 or more assays performed independently.

In our previous research, RNA-Seq analysis of ICGC PDAC-AU specimens and gene set enrichment analysis (GSEA) were carried out [15]. STAT3-induced genes had significant associations with IGFBP2, indicating IGFBP2′s strong correlation with STAT3 signaling. In the same database, we selected patient samples in the top- and bottom-20th percentile of STAT3 expression to identify potential links to immune regulation. We found that PDAC patient tumors with high STAT3 had significantly higher expression of IDO (*p* = 0.014, Figure 4A), a known inhibitor of T cell proliferation and inducer of Treg differentiation. Therefore, we hypothesized that IGFBP2 increases the expression of IDO by activating STAT3 signaling in PDAC, thereby inducing Treg differentiation and promoting tumor progression.

To test this hypothesis, IGFBP2 and IDO amounts in specimens from 50 PDAC patients were assessed by IHC. The results revealed that high IGFBP2 amounts had a positive correlation with IDO signal intensity (Figure 4A). These findings suggested IGFBP2 might control lymphocyte differentiation via the STAT3/IDO pathway. qRT-PCR analysis revealed that IGFBP2 levels were significantly associated with IDO mRNA amounts in clinical tumor samples (*p* = 0.0001, R^2^ = 0.6690; Figure 4B). Cell suspensions from fresh human PDAC tissues, assessed for IGFBP2 expression by IHC, were analyzed by flow cytometry for IDO. PDAC samples with elevated IGFBP2 expression had markedly elevated percentages of IDO^+^ cells (Figure 4C), which is consistent with our IHC (Figure 1A) and qRT-PCR (Figure 4B) results. Furthermore, the levels of tryptophan and its IDO-generated degradation product, kynurenine, were evaluated by ELISA in the culture supernatants of cells generated from fresh human PDAC tissues. We detected decreased tryptophan and elevated kynurenine in IGFBP2-high tumors, commensurate with increased IDO activity (Figure 4D).

To test if IGFBP2 acts upstream of STAT3/IDO signaling, Western blotting was carried out to examine STAT3 activation in lysates from siR-ctrl-transfected and IGFBP2-knockdown (siR-BP2-1 and -2) MDA-PATC53 cells. IGFBP2 silencing downregulated pSTAT3 without affecting total STAT3 expression. IDO expression was also downregulated in IGFBP2 knockdown cells, supporting impairment of STAT3 activation and downstream gene expression. Conversely, IGFBP2 overexpression in MDA-PATC148 BP2 cells resulted in upregulated pSTAT3 and IDO, in comparison with MDA-PATC148 EV cells expressing low levels of endogenous IGFBP2 (Figure 4E). The impact of IGFBP2 on IDO expression was evident at the transcriptional level (*p* < 0.001; Figure 4F).

### 3.5. IGFBP2 Modifies T Cell Differentiation by Inducing IDO Expression in Human PDAC Cells

**Figure 5:**
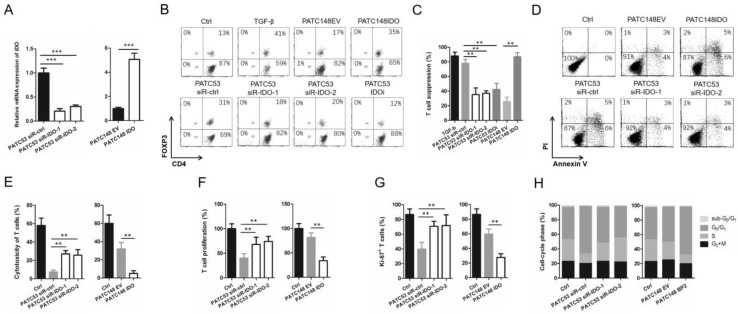
IGFBP2 alters T cell differentiation by inducing IDO expression in human PDAC. (**A**) Relative IDO mRNA expression levels of different PDAC cell lines were assessed by qRT-PCR, normalized to GAPDH (*** *p* < 0.001 by ANOVA for PATC53 or the Student’s *t*-test for PTAC148). (**B**) Flow cytometry analysis of human CD4^+^ T cells cultured without or with TGF-β or co-cultured with PDAC cell lines differentially expressing IDO for CD4^+^FOXP3^+^ Tregs. (**C**) Activated CD8^+^ T cells were co-cultured with the Tregs inducted by TGF-β or PDAC cell lines to verify their T cell suppressive function (** *p* < 0.01 by ANOVA for PATC53 or the Student’s *t*-test for PTAC148). (**D**) Human CD8^+^ T cells co-cultured with PDAC cell lines differentially expressing IDO were assessed flow-cytometrically for apoptotic CD8^+^ T cells. (**E**–**H**) Cytotoxicity, proliferation, Ki-67, and cell cycle analysis was performed on the same CD8^+^ T cells as in (**D**) (** *p* < 0.01 by ANOVA for PATC53 or the Student’s *t*-test for PTAC148). Data are mean ± SD from 3 or more assays performed independently.

Since IGFBP2 regulates IDO expression and alters T cell differentiation, we further investigated whether IDO affects the differentiation of T cells. Here, we established 2 pairs of patient-derived PDAC cell lines with differential expressions of IDO. IDO was overexpressed in MDA-PATC148 cells (low endogenous IGFBP2) and knocked down in MDA-PATC53 cells (elevated endogenous IGFBP2) with two siRNAs (siR-IDO-1 and -2) and included a drug treatment group using the IDO inhibitor Indoximod (100 µM; no. 452483; Sigma). QRT-PCR revealed significantly decreased IDO expression in MDA-PATC53 siR-IDO-1 and -2 after siRNA transfection. Furthermore, more upregulated *IDO* expressions were detected in PATC148 IDO than PATC148 EV (Figure 5A). 

In our model of T cell differentiation, MDA-PATC148-IDO overexpression cells increased the percentages of CD4^+^Foxp3^+^ Tregs compared with MDA-PATC148-EV cells, approaching the levels achieved by TGF-β (Figure 5B). Alternatively, MDA-PATC53 PDAC siR-IDO-1 and -2 cells reduced the percentages of Tregs in comparison with elevated IGFBP2/IDO MDA-PATC53 siR-ctrl cells, being intermediate to the reduction observed for the IDO inhibitor (Figure 5B).

We further co-cultured activated CD8^+^ T cells with the Tregs inducted by TGF-β or PDAC cell lines mentioned above to verify their T cell suppressive function. The results revealed that Tregs induced by IDO silenced (MDA-PATC53 siR-IDO-1 and -2) or inhibited (MDA-PATC53 IDOi) PDAC cells significantly impaired the T cell suppressive function in comparison with Tregs induced MDA-PATC53 siR-ctrl cells with high endogenous IDO. Conversely, MDA-PATC148 IDO cell-induced Tregs showed stronger T cell suppressive function, compared with Tregs induced by MDA-PATC148 EV cells endogenously expressing low IDO (*p* < 0.01; Figure 5C).

Finally, we co-cultured activated CD8^+^ T cells with the IDO-modulated PDAC cell lines to assess T cell function. Our results revealed that MDA-PATC148 IDO overexpression cells induced T cell apoptosis, inhibited cytotoxicity in T cells, and reduced T cell proliferation by promoting G1 arrest, compared with low IDO MDA-PATC148 EV control cells. Conversely, silencing IDO in MDA-PATC53 PDAC cells increased the cytotoxicity of T cells, promoted T cell expansion, and reduced G1 arrest in comparison with elevated endogenous IDO MDA-PATC53 siR-ctrl (Figure 5D–H). Jointly, these IDO findings corroborated IGFBP2 data obtained in parallel experiments, strongly implicating an inter-relationship of IGFBP2 and IDO in promoting an immunosuppressive microenvironment in PDAC.

### 3.6. IGFBP2 Promotes IDO Production in Human PDAC via STAT3 Signaling

**Figure 6:**
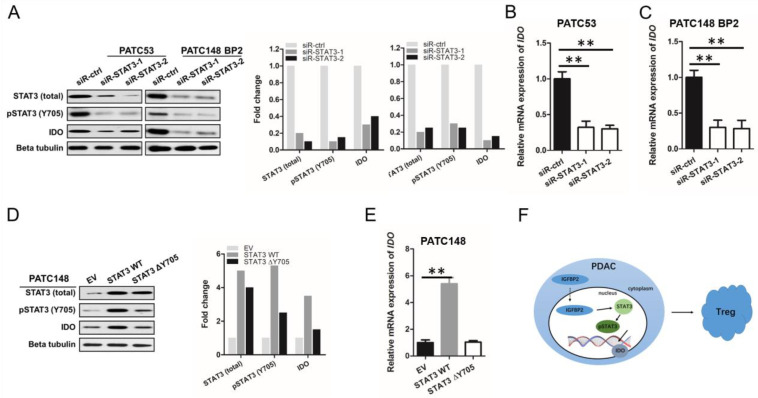
IGFBP2 induces IDO production in human PDAC cells via STAT3 signaling. (**A**) Western blot of high endogenous IGFBP2 MDA-PATC53 cells or IGFBP2-overexpressing PATC148 BP2 after transfection with negative control (siR-ctrl) and STAT3 (siR-STAT3-1 and -2) siRNAs, respectively. STAT3, pSTAT3, and IDO protein amounts were assessed by Western blot, with beta tubulin as a reference protein. IDO mRNA amounts (**B**,**C**) in the above cells were assessed by qRT-PCR, with GAPDH as a reference gene (** *p* < 0.01 by ANOVA). (**D**) Low endogenous IGFBP2 MDA-PATC148 cells underwent transfection with wild type and Y705-mutant STAT3 plasmids, respectively. The protein levels of STAT3, pSTAT3 and IDO were assessed by Western blot, with beta tubulin as a reference protein. IDO mRNA amounts (**E**) in the latter cells were determined by qRT-PCR, with GAPDH utilized for normalization (** *p* < 0.01 by ANOVA). Data are mean ± SD from 3 or more assays performed independently. (**F**) IGFBP2 promotes tumor progression via alternative polarization of macrophages in PDAC in a STAT3 pathway-dependent manner.

To confirm that STAT3 signaling controls the IGFBP2-induced immunosuppressive microenvironment in PDAC cells, gain- and loss-of-function assays were performed. Firstly, we silenced STAT3 (using siR-STAT3-1 and -2) in high endogenous IGFBP2 MDA-PATC53 cells and elevated endogenous IGFBP2 MDA-PATC148 BP2 cells, respectively. Immunoblot showed starkly reduced STAT3, pSTAT3, and IDO amounts after siRNA transfection (Figure 6A). qRT-PCR also revealed significantly decreased IDO expression (Figure 6B,C). We next generated MDA-PATC148 cells with stable overexpression of wild-type or Y705-mutated STAT3. Overexpressing wild-type STAT3 markedly upregulated IDO, while overexpressing mutant STAT3 had no effect (Figure 6D,E).

Jointly, the above findings indicated IGFBP2 promoted STAT3 transcriptional activity in PDAC, upregulating IDO, a STAT3 downstream effector. Additionally, IDO induces the differentiation of Tregs and promotes tumor progression. The proposed mechanistic steps are provided in Figure 6F.

## 4. Discussion

In this study, clinical and experimental findings highlighted the clinical relevance of IGFBP2 in immunoregulation in PDAC. Cases with tumors exhibiting elevated IGFBP2 had greater accumulation of Tregs in the TME, which was reflected by reduced PDAC patient survival. We further demonstrated that IGFBP2 augmented IDO expression by activating STAT3 signaling in PDAC, thereby inducing Treg differentiation and promoting tumor progression. A major finding of the current work is that IGFBP2 was shown to function as an immunomodulator by inducing the differentiation of Tregs in the TME of PDAC. Identification of the role of IGFBP2 with respect to immunoregulation in PDAC may open new immunotherapeutic avenues, including transforming PDAC from an immuno-cold tumor into a hot tumor.

STAT3 is considered a major driver of immune evasion and tumor progression. The STAT3 signaling pathway activated multiple immunosuppressive cells to maintain an immunosuppressive TME [25,26]. STAT3 is a FOXP3 inducer that promotes the immunosuppressive function of Tregs. STAT3 activation in cancer cells enhances the expression of IDO, which plays immunoregulatory roles in T-cells by regulating tryptophan metabolism [27]. IDO-derived tryptophan catabolites inhibit cytotoxic T cell proliferation and promote Treg differentiation [28].

Multiple modulators control STAT3 activation. Our previous research established that IGFBP2 potentiates STAT3 transactivation activities by increasing nuclear EGFR amounts in glioma. Our recent work also revealed IGFBP2 drives the EMT and reflects poor prognosis in PDAC. Here, IGFBP2 augmented STAT3 transactivation activities in PDAC and increased the expression of IDO, which is a demonstrated STAT3 downstream gene [29]. IDO upregulation in PDAC activates STAT3 in immune cells. Thus, STAT3 is an important player in inflammation-associated malignancies, including PDAC, and IGFBP2 initiates this feedforward loop.

Tumors can promote an immunosuppressive microenvironment through multiple mechanisms and ultimately evade immunosurveillance, including checkpoint suppression of T-cell activation and IDO upregulation [30]. IDO empowers the efficacies of chemotherapeutics, radiation therapy, cancer vaccines, and immune checkpoint inhibitors without additional side effects [31,32]. Combining small-molecule IDO inhibitors with checkpoint inhibitors in cancer treatment may substantially improve patient outcomes. Exciting results have been reported by clinical trials combining the IDO inhibitor epacadostat and PD1 antibodies for treating skin, head and neck, lung, renal, and urothelial cancers. For example, the definitive phase III trial of melanoma that assessed combined epacadostat and pembrolizumab, a PD1 antibody, failed. This failure could be partly due to both the IDO inhibitor and PD1 antibody focusing on the awakening of cytotoxicity T cells. Indeed, tumors mediate immunosuppression not only by inducing T cell anergy but also by recruiting multiple immunosuppressive cells, including M2 macrophages, Tregs, and MDSCs, which would impede T cell recruitment first and then confer an immune-cold condition to the tumor [33]. 

IGFBP2 is considered a multifunctional oncogenic protein with tumor promoting functions through multiple signaling pathways in many cancers. We have demonstrated that IGFBP2 regulates EMT and metastasis by NF-κB signaling in our previous research. In addition, IGFBP2 increases the nuclear accumulation and activation of EGFR to promote STAT3 transcriptional activity and upregulate its downstream genes. Growing evidence implicates IGFBP2 in playing a vital role in immune modulation. Numerous findings indicate IGFBP2 always leads to an immunosuppressive microenvironment and tumor progression. In malignant melanoma, IGFBP2 upregulates the PD-L1 expression and contributes to the immune evasion of cancer cells from host immunosurveillance [34]. Accumulation of IGFBP2 can be found in activated microglia/macrophages, which are important glioma-infiltrating immune cells, and this may contribute to glioma development [34]. The IGFBP2-positive macrophages/microglia frequently accumulate near the focal necrosis areas of glioma, suggesting an important immune action for IGFBP2 in glioma [35]. In glioblastoma, IGFBP2 correlates with an increase of M2 macrophages and a decrease of B cells and cytotoxicity T cells [36]. Bioinformatic analysis for gene expression profiles of 2447 glioma samples demonstrated that IGFBP2 has immunosuppressive activities in glioblastoma (GBM), and IGFBP2 is positively correlated with immunosuppressive molecules’ expression, including VEGFA, CHI3L1, ANXA1, TNFRSF1A, LGALS3, TIMP1 and LGALS1 [37]. Furthermore, we revealed that IGFBP2 induced alternative polarization of macrophages and promoted PDAC progression through the STAT3 signaling pathway. The enhancement of Treg differentiation by IGFBP2 that was revealed in this research further extends the immune-modulated functions of IGFBP2 in the PDAC microenvironment. As a single checkpoint inhibitor, targeting STAT3/IDO or EGFR/COX2 achieved only a modest effect in the treatment of PDAC; the combined blockade of multiple checkpoint pathways by targeting IGFBP2 should be a promising strategy to better control PDAC.

In summary, IGFBP2 induces PDAC progression by enhancing Treg differentiation, which is controlled by STAT3 signaling that induces IDO in the TME. This implicates that IGFBP2 could be targeted as an efficient way for overcoming PDAC resistance to immunotherapeutic agents in clinics.

## Data Availability

The original data reported in this study are included in the article/Supplementary Material. Further inquiries can be directed to the corresponding authors.

## Supplementary Materials

The following supporting information can be downloaded at: https://www.mdpi.com/article/10.3390/jpm12122005/s1, Table S1: Information about the siRNAs tested, Table S2: Data of the 91 patients from the ICGC PADC-AU cohort, and their expression levels (FPKM) of related genes.
