# Supplementary material for: IGFBP2 Drives Regulatory T Cell Differentiation through STAT3/IDO Signaling Pathway in Pancreatic Cancer
Source: J Pers Med. 2022 Dec 3;12(12):2005. doi: 10.3390/jpm12122005 (PMC9785430; doi:10.3390/jpm12122005)
Supplement: Supplementary file 1 [file jpm-12-02005-s001.zip › Supplementary Table S1.pdf]

Gene Symbol	Species	siRNA Type	Manufacturer	Product Number	siRNA ID	Sequence Start
IGFBP2	Human	Rosetta Predictions	Sigma	NM_000597	SASI_Hs02_00302878	926
					SASI_Hs01_00039595	1032
IDO1	Human	Rosetta Predictions	Sigma	NM_002164	SASI_Hs01_00237226	638
					SASI_Hs01_00237227	1100
STAT3	Human	Rosetta Predictions	Sigma	NM_139276	SASI_Hs01_00061860	1029
					SASI_Hs01_00061861	1981
