# Supplementary material for: IGFBP2 Drives Regulatory T Cell Differentiation through STAT3/IDO Signaling Pathway in Pancreatic Cancer
Source: J Pers Med. 2022 Dec 3;12(12):2005. doi: 10.3390/jpm12122005 (PMC9785430; doi:10.3390/jpm12122005)
Supplement: Supplementary file 1 [file jpm-12-02005-s001.zip › Supplementary Table S2.pdf]

ICGC_Cohort

sample	time	censor	sex	age	IGFBP2	STAT3	IDO1	IL10	NOS2	CD4	MSR1	FOXP3	TREM2	CD40	ARG1	CCR7	PPARG	IL2RA	MKI67	CD163
DO32829	1874	deceased	female	78	62.34	93.42	4.83	0.41	0.27	13.23	41.15	0.38	14.25	14.01	0	0.33	8.32	2.05	12.64	29.97
DO32860	1259	deceased	male	57	58.87	70.12	1.32	0.75	0.06	15.57	45.75	0.39	17.3	7.9	0	1.01	45.43	7.02	6.78	15.82
DO32863	715	deceased	male	60	34.04	67.94	1.45	0.35	0.11	24.9	109.06	0.24	19.51	6.48	0.06	0.51	55.35	3.47	5.94	27.88
DO32875	348	deceased	male	69	30.27	81.95	36.35	0.58	0.25	11.24	23.14	0.74	7.12	15.8	0.02	1.06	27.16	6.16	9.35	78.64
DO32878	260	deceased	female	69	71.72	52.1	0.5	0.35	0.04	5.15	17.18	0.17	5.49	6.86	0.07	0.26	23.45	1.7	6.91	13.01
DO32887	359	deceased	female	79	91.14	45.84	0.3	2.5	1.39	3.49	6.76	0.19	2.55	6.86	32.3	0.23	23.18	1.51	19.18	18.5
DO32900	768	deceased	male	51	94.93	68.81	2.74	0.61	0.19	11.77	17.01	0.4	3.34	6.73	0.06	1.43	26.1	2.09	7.36	14.89
DO32936	552	deceased	male	68	1.57	41.25	0.67	0.06	0.02	1.25	0.1	0.04	0.04	4.06	0	0.08	64	0.01	13.04	0
DO33091	142	deceased	male	56	0.88	124.9	0.03	0.17	0.02	0.03	0.37	0.02	0	9.11	0	0	0.24	0	6.23	0.01
DO33128	423	deceased	male	72	82.99	93.96	5.92	0.95	0.12	18.97	50.94	0.53	16.18	8.28	0	0.97	25.07	5.9	14.6	45.11
DO33168	8	deceased	female	80	34.495	63.635	3.315	2.145	0.185	12.28	19.685	1.255	4.58	8.25	0.025	1.755	1.83	4.755	6.225	16.29
DO33256	476	deceased	female	65	0	19.14	0.21	0.09	0	0.14	0.07	0	0	7.28	0	0	23.82	0	3.52	0
DO33336	451	deceased	female	67	10.07	65.56	1.73	1.14	0.16	7.07	12.35	0.48	3.41	6.3	0.09	6.27	51.65	1.37	10.99	5.9
DO33344	361	deceased	male	70	58.5	69.14	1.98	0.44	0.37	7.16	14.53	0.32	5.3	12.6	0.08	0.42	12.06	2.11	9.31	7.51
DO33368	98	deceased	male	81	8.69	44.87	5.07	2.12	0.12	8.92	42.36	0.29	10.88	4.15	0.12	0.49	3.78	2.73	10.17	73.43
DO33376	909	alive	female	62	39.25	66.22	1.49	0.51	0.12	6.26	21.8	0.47	3.61	5.86	0.17	0.39	14.23	1.66	5.67	18.56
DO33392	1014	alive	male	70	16.52	105.52	0.29	0.43	0.08	3.46	3.73	0.17	1.74	1.31	0	0.51	15.34	0.95	6.73	9.74
DO33400	1043	alive	female	76	4.67	48.07	0.6	11.89	0.1	2.19	5.25	0.04	4.03	2.94	0	0.03	19.11	0.52	4.63	4.17
DO33408	909	alive	female	68	95.84	65.87	1.16	0.92	0.08	19	48.15	0.5	20.31	7.11	0.09	0.96	15.08	8.78	4.73	29.96
DO33472	388	deceased	female	75	180.02	66.72	1.12	1	0.06	5.58	9.43	0.18	3.98	14.21	0	0.12	9.58	1.23	12.37	5.24
DO33480	1054	alive	male	52	62.88	52.96	0.65	1.17	0.14	7.61	24.78	0.03	13.21	2.54	0	0.21	11.85	1.32	2.19	9.78
DO33488	844	alive	male	61	207.33	96.85	1.76	0.7	0.34	7.65	20.32	0.39	5.83	1.81	0.02	0.4	12.86	0.69	17.64	15.03
DO33512	499	deceased	female	NA	19.33	55.2	0.43	0.2	0.12	8.1	17.68	0.28	6.1	10.67	0.07	0.17	74.01	0.95	6.71	8.94
DO33528	343	deceased	male	36	0.58	20.15	7.14	0.14	0.01	0.17	0.05	0	0	4.17	0	0	32.5	0	6.32	0.02
DO33544	412	deceased	female	83	26.66	66.97	5.55	0.39	0.47	13.33	11.71	0.8	5.12	8.84	0.09	2.24	15.45	2.45	9.52	25.14
DO33552	873	alive	female	70	7.01	59.01	0.53	0.05	0.1	4.62	9.9	0.17	2.67	4.22	0.09	0.27	13.72	2.33	9.78	10.78
DO33600	854	alive	female	77	16.26	75.85	5.86	0.7	0.29	7.07	14.26	0.49	7.2	8.38	0	0.91	19.18	2.05	3.12	13.1
DO33632	1105	deceased	male	67	0.08	22.55	0.09	0.1	0	0.05	0.03	0	0	6.77	0	0	34.34	0	6.51	0
DO33656	164	deceased	female	76	0.4	7.71	0	0.09	0	0	0	0	0	0	0	0	0.66	0	1.55	0
DO33984	847	alive	male	64	61.4	91.03	6.73	1.05	0.74	20	37.74	0.71	21.29	6.86	0.05	2.56	8.8	3.39	12.21	66.25
DO34240	236	deceased	male	72	52.38	80.62	0.52	0.52	0.44	11.37	57.18	0.33	11.45	6.16	2.19	0.34	8.36	4.53	4.34	35.23

DO34264	1144	deceased	female	63	20.88	61.58	1.64	0.35	0.06	6.98	11.5	0.24	4.73	6.73	0.02	0.92	10.22	0.83	3.82	10.61
DO34288	220	deceased	female	83	95.64	77.1	1.55	0.38	2.35	7.75	21.43	0.28	7.73	8.53	0.16	0.4	35.23	1.09	16.3	9.67
DO34312	226	deceased	female	58	9.53	57.8	8.19	0.52	0.03	6.37	27.93	0.28	7.4	3.9	4.16	0.26	16.82	3.01	5.42	12.89
DO34336	743	alive	female	70	3.31	3.97	0	17.61	0	1.52	0.3	1.17	0.52	1.27	0	2.82	3.06	0	0.88	0.24
DO34368	537	deceased	female	64	29.47	66.74	1.3	0.4	0.69	10.87	11.61	0.32	4.07	5.72	0.08	1.14	75.81	2.4	11.39	12.99
DO34376	179	deceased	male	61	1.73	42.31	3.32	0.08	0	0.64	0.05	0.02	0.02	5.97	0.02	0.01	151.99	0	7.98	0.01
DO34432	400	deceased	female	82	44.05	90.65	1.1	0.72	0.12	17.42	13.56	0.53	12.79	15.89	0.06	0.58	4.24	2.97	6.57	17.4
DO34448	719	deceased	female	79	77.62	84.07	1.02	0.99	0.12	12.34	61.24	0.26	20.44	4.2	0.29	0.1	11.93	0.38	11.44	19.39
DO34504	257	deceased	male	37	41.58	93.96	1.45	0.56	0.05	15.22	17.64	0.61	7.16	11.28	0.2	0.93	19.63	3.81	7.24	34.39
DO34600	537	deceased	male	77	84.14	71.51	2.72	0.36	0.16	5.02	6.36	0.74	3.23	3.9	0.03	1.41	7.62	3.57	6.31	14.35
DO34608	709	deceased	female	72	78.48	103.94	5.01	0.5	0.12	7.83	13.32	0.36	8.15	7.34	0.04	1.8	39.6	1.4	11.36	10.6
DO34616	666	alive	female	68	60.16	55.68	0.13	0.2	0.29	2.21	2.88	0.06	1.12	1.8	0.15	0.24	46.78	0.84	15.2	1.96
DO34640	287	deceased	female	49	10.4	62.96	8.92	0.22	0.07	10.05	24.47	0.62	6.21	16.01	0.05	2.36	20.88	3.54	16.7	15.29
DO34656	834	alive	female	61	22.67	53.15	1.36	0.38	0.16	10.2	13.84	0.56	8.8	12.55	0.03	0.79	27.47	4.41	11.6	36.06
DO34680	99	deceased	male	75	34.89	105.52	2.03	0.71	0.05	5.31	10.13	0.25	2.9	7.12	0	0.22	28.83	0.71	9.35	6.65
DO34696	689	alive	male	75	31.36	92.25	71.52	0.44	0.38	12.51	26.78	0.47	10.45	17.21	0	0.73	57.65	3.14	6.88	19.15
DO34720	555	alive	male	68	9.65	106.85	4.03	1.45	0.08	12.09	41.36	0.73	14.23	7.33	0.05	0.69	31.01	2.36	13.09	22.66
DO34728	508	alive	female	77	134.87	94.16	7.18	0.8	0.15	18.25	43.83	0.74	18.31	6.15	0.04	0.97	2.75	14.15	6.95	69.78
DO34736	203	deceased	female	78	40.69	75.26	61.66	0.5	2.27	12.82	20.6	0.76	4.99	5.71	0.13	2.31	13.25	3.89	14.33	15.51
DO34785	721	alive	female	67	36.24	75.09	1.4	0.31	0.14	8.7	17.08	0.27	6.41	9.8	0.06	0.83	69.55	2.51	9.14	8.87
DO34793	427	deceased	male	56	52.96	90.39	2.19	1.03	0.06	8.7	14.49	0.78	5.02	1.82	0	1.16	28.6	4	3.08	17.58
DO34801	626	alive	male	60	46.59	66.07	2.34	0.61	1.41	11.88	11.19	0.19	4.68	2.59	0.08	0.54	31.79	1.08	8.24	5.01
DO34809	297	deceased	female	74	42.87	41.37	39.67	0.65	1.58	6.87	12.13	0.45	2.84	3.04	0.23	1.23	13.97	2.28	11.1	9.67
DO34817	540	alive	female	72	67.64	91.52	12.6	0.35	0.05	14.08	11.07	0.38	7.33	5.58	0.08	2.26	23.8	2.4	16.88	16.03
DO34849	272	deceased	female	86	58.84	51.11	0.68	1.69	0.79	12.82	45.12	0.81	13.17	14.67	0.05	0.21	24.79	2.33	11.19	21.11
DO34905	1142	alive	male	64	24.51	75.61	26.04	0.5	0.25	2.93	8.46	0.36	1.31	11.51	0.07	0.1	36.99	1.44	14.79	6.04
DO34945	189	deceased	male	68	15.2	47.25	0.38	2.97	0.09	8.76	42.08	0.25	10.71	12.15	0.21	0.28	25.65	4.15	8.06	93.96
DO34961	632	deceased	male	71	3.84	35.11	0.77	0.65	0.01	4.14	17.04	0.07	6.61	1.36	0.06	0.07	1.65	0.8	7.16	5.39
DO49074	168	deceased	male	49	114.4	38.56	0.04	1.9	0	0.57	3.95	0.2	0.7	4.85	0.08	0.03	22.16	0.25	22.96	1.74
DO49076	480	deceased	male	64	185.04	48.66	7.2	0.76	0.07	11.91	19.3	0.57	7.17	2.75	0	5.26	2.16	2.16	2.46	17.16
DO49078	465	deceased	male	79	214.7	72.33	6.03	0.69	0.25	13.8	21.79	0.97	5.82	4.59	0	3.31	18.42	1.91	13.52	13.44
DO49079	332	deceased	male	50	7.68	58.38	5.31	0.85	0.05	4	11.47	0.5	4.74	10.25	0.1	0.13	3.19	6.56	6.17	26.1
DO49080	83	alive	male	60	64.09	93.66	13.06	1.2	0.2	8.92	22.08	1.22	4.96	9.46	0.17	0.66	6.01	6.98	10.89	24.65

DO49087	260	deceased	female	68	67.05	53.29	0.52	1.46	0.35	6.64	11.69	0.32	2.07	3.24	0.72	1.09	12.73	1.32	17.42	23.07
DO49090	152	deceased	female	77	12.64	43.69	0.85	0.75	0.03	4.57	15.46	0.06	5.31	3.57	0.05	0.12	30.46	3.72	5.52	15.6
DO49105	445	deceased	male	59	45.63	32.37	0	0.06	0	0.01	0	0.01	0	5.1	0	0.01	43.84	0	6.79	0
DO49113	413	alive	female	46	20.32	76.39	3.84	0.59	0.21	8.58	21.27	0.36	4.01	4.01	0	0.29	21.78	3.58	20.7	12.66
DO49127	392	alive	female	77	59.05	62.21	1.09	0.85	0.1	12.48	25.7	0.84	6.16	8.62	0	1.41	43.4	4.17	5.23	16.16
DO49129	337	alive	male	50	55.13	80.81	1.84	0.37	0.17	7.14	19.46	0.29	5.01	8.94	0	0.49	55.47	2.03	6.88	9.42
DO49130	351	alive	female	76	45.33	53.95	6.9	0.95	0.12	8.9	20.66	1.03	4.56	2.87	0.11	0.65	29.7	1.89	16.09	13.11
DO49133	188	alive	female	55	67.76	61.31	2.15	0.45	0.16	14.58	15.66	0.41	7.48	6.95	0	2.14	28.07	5.87	14.54	35.83
DO49135	270	alive	male	65	47.89	76.29	0.83	0.31	0.53	6.86	15.03	0.09	5.25	11.6	0.15	0.28	13.91	5.52	5.85	24.29
DO49137	232	alive	female	67	54.51	86	2.86	0.49	0.27	14.5	17.8	0.69	5.14	3.78	0.09	1.71	20.26	4.13	13.44	33.19
DO49138	230	deceased	male	74	17.46	100.08	2.33	0.62	0.05	8.79	23.61	0.45	6.69	6.28	0.04	0.45	14.92	3.4	6.51	7.83
DO49164	272	alive	male	70	81.79	75.36	2.58	0.48	0.3	10.4	31.97	0.22	8.98	14.57	0.13	1.28	69.99	3.31	5.14	21.97
DO49166	222	alive	female	40	8.89	79.04	1.72	0.58	0.06	10.06	26.89	0.67	5.45	8.44	0.05	0.71	21.44	6.54	15.94	27.41
DO49168	149	alive	male	58	39.71	74.8	6.06	1.18	0.62	14.13	43.71	0.75	11.47	8.91	0.09	8.06	7.85	4.67	9.99	20.4
DO49170	197	alive	male	67	74.64	78.9	4.17	0.91	0.12	23.54	42.91	0.47	10.21	13.28	0.04	0.95	10.65	3.39	4.19	31.16
DO49172	1021	deceased	female	60	50.79	77.48	0.58	1.64	0.01	3.8	10	0.34	2.89	9.62	0.06	0.15	43.96	1.79	21.63	4.22
DO49175	414	deceased	male	68	2.04	49.76	1.04	2.85	0.02	0.69	4.12	0	0.09	1.73	0.12	0.22	31.84	0.52	6.58	2.84
DO49178	27	deceased	male	58	13.32	72.06	4.63	0.9	0.23	9.14	31.51	0.41	6.38	9.07	0	0.61	12.41	1.22	22.78	20.63
DO49181	1534	deceased	male	77	31.95	68.38	0.43	1.12	0.13	4.77	12.7	0.26	4.7	6.31	0	0.12	17.56	0.51	8.08	7.59
DO49183	429	deceased	female	51	17.41	81.18	3.27	0.79	0.21	4.88	13.66	0.38	1.68	6.23	0	0.57	32.62	2.69	15.7	12.58
DO49184	455	deceased	male	56	32.54	58.09	0.62	2.55	0.28	14.37	23.32	0.65	5.34	11.91	0	0.26	12.87	11.07	23.46	30.12
DO49185	179	alive	male	55	24.24	87.15	1.36	0.93	0.2	7.31	26.6	0.63	4.23	5.52	0.05	0.93	44.94	4.38	12.24	22.92
DO49193	1	deceased	male	60	23.22	70.87	2.32	0.3	0.04	12.19	21.72	1.01	4.75	12.22	0	0.93	40.92	4.16	8.1	8.31
DO49198	251	deceased	female	61	15.18	71.87	1.3	1.18	0.08	2.55	7.76	0.27	0.38	5.72	0	0.17	10.22	2.9	6.47	5.06
DO49199	1095	deceased	male	52	26.32	81.82	2.29	1.23	0.12	10.83	21.71	1.94	2.74	7.55	0	0.96	11.97	2.3	16.63	5.92
DO49201	388	deceased			180.02	66.72	1.12	1	0.06	5.58	9.43	0.18	3.98	14.21	0	0.12	9.58	1.23	12.37	5.24
DO49204	156	deceased	male	67	79.57	65.36	4.25	0.76	0.16	9.74	24.49	0.39	4.96	15.48	0.07	8.19	4.17	4.8	18.04	26.74

Immune_Marker

**T-reg**	**CD8-T**
CD25	CD3D
CD4	CD3E
IL2RA	CD3G

FOXP3	CD8A
MKI67	CD8B
